# The Total Score of the Level of Personality Functioning Scale Is Empirically and Theoretically Well‐Justified: A Reply to Zavlis (2025)

**DOI:** 10.1002/pmh.70049

**Published:** 2025-11-05

**Authors:** Johannes Zimmermann, André Kerber, Susanne Hörz‐Sagstetter, Christopher J. Hopwood, Ludwig Ohse

**Affiliations:** ^1^ Department of Psychology University of Kassel Germany; ^2^ Division of Clinical Psychological Intervention Freie Universität Berlin Berlin Germany; ^3^ Department of Clinical Psychology and Psychotherapy Psychologische Hochschule Berlin Berlin Germany; ^4^ University of Zürich Zürich Switzerland; ^5^ Department of Psychology MSB Medical School Berlin Berlin Germany

We thank Zavlis (2025) for his commentary on our recently published paper (Ohse et al. 2025). In that paper, we examined the empirical convergence between Kernberg's model of personality organization (Kernberg and Caligor 2005) and the Alternative DSM‐5 Model for Personality Disorders (AMPD; American Psychiatric Association 2013), including the constructs of personality functioning (PF; Bender et al. 2011) and pathological personality traits. Using exploratory bifactor models, we found that measures of Kernberg's model and the AMPD strongly converged regarding both general and specific personality pathology. Zavlis (2025) criticized our methodological approach, arguing it rests on the “implicit assumption” that PF is a “unitary entity.” He conducted secondary analyses of our published data to test whether PF “is truly unitary” and concluded that PF “is better understood both theoretically and psychometrically in terms of two distinct domains.”

We concur with Zavlis (2025) that a perspective on PF that considers the domains of self‐ and interpersonal functioning separately may offer valuable insights (e.g., Zimmermann 2022) and could be linked to established theories of personality development (e.g., Blatt 2008). Indeed, this view is consistent with empirical results showing that distinct albeit highly correlated factors of self and interpersonal functioning can be identified in many measures of PF (Zimmermann et al. 2023). However, we contend that the commentary has several weaknesses, including its use of ambiguous terminology, a lack of transparency in its secondary analyses, and a failure to engage with our original statistical model and theoretical rationale. Below, we outline specific arguments that challenge the assertion that a two‐factor model of PF is superior.

First, the primary evidence against a “unitary” view of PF is a model comparison that pits a two‐factor model against a one‐factor model, using the 12 subscales of the Level of Personality Functioning Scale—Self‐Report (LPFS‐SR; Morey 2017) as indicators. This reveals that Zavlis (2025) implicitly defines the term “unitary entity” (which seems rather vague and has no established basis in the psychometric literature) as a strictly unidimensional construct. To our knowledge, no author posits that PF can be fully captured by a single latent factor with no residual specificity. While strict unidimensionality may be a reasonable assumption for narrow personality facets, it is an unrealistic standard for broader and more complex constructs such as PF (e.g., Revelle and Condon 2025). This model comparison, therefore, tests a straw man. Rejecting this overly simplistic model based on poor fit indices is an unsurprising result that does not challenge our original, more nuanced model.

Second, Zavlis (2025) is not transparent about key specifications of the two‐factor model presented as superior. Inspecting the provided code, we found that the two factors were highly correlated (*r* = 0.80), a finding that itself supports a strong general factor. More concerningly, the model was not a standard correlated factors model but one that included a cross‐loading and five correlations between residuals. It is reasonable to infer that these post hoc modifications were made to improve model fit and reduce the factor correlation, as a standard two‐factor model fit the data considerably worse (scaled *χ* ^2^[53] = 170.95, CFI = 0.929, RMSEA = 0.090, SRMR = 0.051) and yielded an even higher factor correlation (*r* = 0.84). Allowing five correlated residuals is statistically equivalent to specifying five minor specific factors, meaning the specified model actually included seven factors. Presenting the fit of this seven‐factor model as support for a parsimonious and theoretically preferred two‐factor model is misleading. This practice falls short of transparent reporting standards by obscuring the additional theoretical complexity required to make the model fit the data.

Third, Zavlis (2025) does not test his proposed model against the exploratory bifactor model we used in our paper. Bifactor models are uniquely suited to disentangle shared general variance from domain‐specific variance. When using the 12 subscales of the LPFS‐SR as indicators, an exploratory factor analysis with three factors and orthogonal biquartimin rotation demonstrated acceptable fit (scaled *χ* ^2^[33] = 88.45, CFI = 0.966, RMSEA = 0.078, SRMR = 0.026), yielded a strong general factor (explained common variance = 0.84, omega hierarchical = 0.92), with all indicators loading substantially on it (ranging from 0.41 to 0.87). This supports an interpretation of PF as “unidimensional enough” for using the total score (Reise et al. 2013; Revelle and Condon 2025), which is, in our view, compatible with the notion of PF as a “unitary entity.” Furthermore, the more extensive bifactor model presented in our paper showed that PF subscales related to identity and self‐direction formed a specific factor with facets of negative affectivity, while PF subscales related to empathy and intimacy formed a specific factor with facets of detachment and impaired object relations (Ohse et al. 2025). Consequently, the finding Zavlis (2025) presents from his secondary regression analyses—that a “self‐focused domain” is associated with “emotional psychopathology” and an “other‐focused domain” with “social psychopathology”—was already demonstrated in Table 2 of our paper.

Fourth, Zavlis (2025) fails to engage with the theoretical rationale for choosing a bifactor model. Our selection was theoretically grounded in our research question about general versus specific pathology, as both the AMPD and Kernberg's model conceptualize PF as a general continuum of severity distinct from stylistic, trait‐like aspects (Hörz‐Sagstetter et al. 2021; Morey et al. 2022). These models justify the focus on the general factor of PF by the clinical utility of a severity index and the psychodynamically grounded assumption of an underlying dimension of developmental maturity (Hopwood 2025; Kerber et al., 2025). Notably, Zavlis's (2025) rejection of bifactor models also contradicts the logic of the interpersonal circumplex (IPC; Wright et al. 2023), which he cites to support his claim for a two‐factor model. As we noted in our paper (Ohse et al. 2025, 10), modeling interpersonal problems from an IPC perspective requires separating the general severity factor so that agency and communion can emerge as specific factors (Wendt et al. 2019). Taken together, while we agree that general factors can be “abstract psychological entities that lack substantive meaning,” we consider this criticism in the context of PF to be overly broad, uninformed by the underlying theories, and unsubstantiated by the data.

What, then, can be learned from this exchange? The central claim of Zavlis's (2025) commentary—that “personality functioning is not a unitary entity”—is either trivial or false depending on how one defines this ambiguous term. His conclusion that a two‐factor model is superior rests on a flawed comparison to a straw man model and a failure to engage with the relevant statistical and theoretical details. More generally, a serious empirical test between correlated factor and bifactor models should certainly not be limited to a single self‐report measure of PF. From a less agenda‐driven perspective, one could even argue that forcing a definitive choice between these models is unproductive. Both models can be empirically and theoretically justifiable, each highlighting different aspects of the data. Instead of pursuing bold but unsubstantiated claims, we believe the field would be better served by promoting research on PF that is self‐critical, pluralistic, and methodologically rigorous.

## Conflicts of Interest

The authors declare no conflicts of interest.

## Data Availability

The data that support the findings of this study are openly available in OSF at https://osf.io/pcn6t/.
